# The use of cattle *Bos taurus* for restoring and maintaining holarctic landscapes: Conclusions from a long‐term study (1946–2017) in northern England

**DOI:** 10.1002/ece3.5169

**Published:** 2019-04-29

**Authors:** Stephen J. G. Hall, Robert G. H. Bunce

**Affiliations:** ^1^ Estonian University of Life Sciences Tartu Estonia

**Keywords:** cultural landscapes, free‐ranging cattle, high nature value pastoral systems, rewilding, ungulate population dynamics, vegetation monitoring

## Abstract

1. Cattle *Bos taurus* can perform valuable ecological functions in the maintenance of high nature value (HNV) pastoral systems. They have also attracted attention as potentially filling the ecological niches of megaherbivores, notably the extinct aurochs *Bos primigenius*, in rewilding initiatives. Native cattle breeds are recognized under the 1992 Rio Convention as components of biodiversity. They are used in HNV settings, but their conservation as breeds has rarely been an important consideration for their management in these contexts.
2. The Chillingham herd has been kept under minimal management in Chillingham Park (northern England) for several centuries. Chillingham Park is not a rewilding scenario, but the long‐term study of the cattle can be informative for the design of rewilding schemes that involve cattle as megaherbivores. The pastures of the park are species‐rich seminatural grasslands.
3. To 2004, pasture management was influenced by the need to provide herbage for a flock of sheep that was under separate ownership, as well as for the cattle. Surveys of the vegetation conducted in 1979 and 2006–2008 showed a decline of plant species richness (species per 100 m^2^ quadrat) from 33.8 in 1979 to 22.6 in 2006–2008. This was acceptable as the conservation priority has always been the cattle herd. With removal of the sheep from 2004, it became possible to include recovery of plant diversity as a management goal.
4. In 2017, the cattle numbered 111 (64 in 1979). Plant species richness in 2017 had increased to 26.3 species per quadrat. It has therefore been possible at Chillingham both to conserve the cattle herd and to improve plant diversity. While providing basic information of relevance to the management of cattle in free‐ranging situations, this study also suggests a general principle, that the management of pastoral landscapes by native breeds of cattle, can deliver multiple conservation benefits.

## 1 INTRODUCTION

Cattle *Bos taurus* are of acknowledged value in the management of high nature value (HNV) pastoral landscapes and are being used in many European countries, to restore floral and faunal diversity in anthropogenic landscapes (Redecker, Finck, Härdtle, Riecken, & Schröder, 2002). As individual native breeds, they are acknowledged under the Convention on Biodiversity as components of global biodiversity (FAO, 2012) and their conservation is an obligation of signatories to the Convention. For example, in the UK Biodiversity Framework, the conservation of 88 native breeds of livestock (including Chillingham and 24 other cattle breeds) is monitored under the Biodiversity Indicators system (JNCC, 2018).

In addition to the economic value of the breeds as genetic resources, they also have intrinsic or existence values as meriting conservation in their own right independent of potential for economic utilization. The Chillingham herd is an extreme example of this intrinsic value, having been supported for several centuries under the guardianship originally of the Earls of Tankerville and, since 1939, of the Chillingham Wild Cattle Association (CWCA), as a purely charitable activity.

Although known as Wild Cattle, they are descended from husbanded stock, and, while receiving minimal management intervention (culling on the basis of individual welfare, but no castration), the herd is confined in an area of 134 ha and there is winter hay feeding. This cannot be seen as a rewilding operation of any form, but in the absence of truly feral and accessible herds anywhere in the world, it can provide basic biological information of potential use for rewilding scenarios notably those where restoration of food webs is envisaged (“trophic rewilding”: Genes, Svenning, Pires, & Fernandez, 2019; Svenning et al., 2016). Pettorelli et al. (2018) mention (their Table 3) seven sets of specific targets for rewilding initiatives, four explicitly involving the reintroduction of megaherbivores.

Our long‐term ecological study on the historic Chillingham cattle has been conducted in a single locality; it is observational and not experimental, and the cattle have many unique features (which is a major reason why they have been conserved). Nonetheless, it is possible to draw conclusions which are relevant to policy and practice elsewhere, while aspiring to meet the definition (Baumgärtner, Becker, Frank, Müller, & Quaas, 2008) of an ecological case study as “the descriptive, explorative, and prospective study of a concrete real‐world situation, including its practical context and determining factors, for the purpose of generating and testing hypotheses”.

The broader context of this study is the issue of multifunctional conservation, with particular reference to the place of livestock biodiversity in socio‐ecological systems. We believe this is the first long‐term study of such a situation.

The present paper commences with a description of the study area and of the herd, followed by details of its population dynamics. We then summarize the vegetation surveys we made in 1979, 2006–2008, and 2017. Changes in vegetation over the 38‐year period are discussed in parallel with changes in the population dynamics of the cattle since 1946. Finally, we consider implications of this case study for the conservation of pastoral landscapes.

## 2 CHILLINGHAM PARK AND THE CHILLINGHAM CATTLE

Chillingham Park is in northern England (55°31′N, 2°54′W), between 98 and 235 m above sea level, at the boundary of an area of mixed moorland and enclosed farmland. It is one of the very few landscape parks in Britain where the tree and pasture components have both survived almost in their original state (Bunce & Hall, 2013; Hall, 2013). In nearby farmland, most grass fields are lacking in plant species richness (R.G.H. Bunce, unpublished). The plant species richness of Chillingham Park is therefore of both regional and national interest.

**Figure 1:**
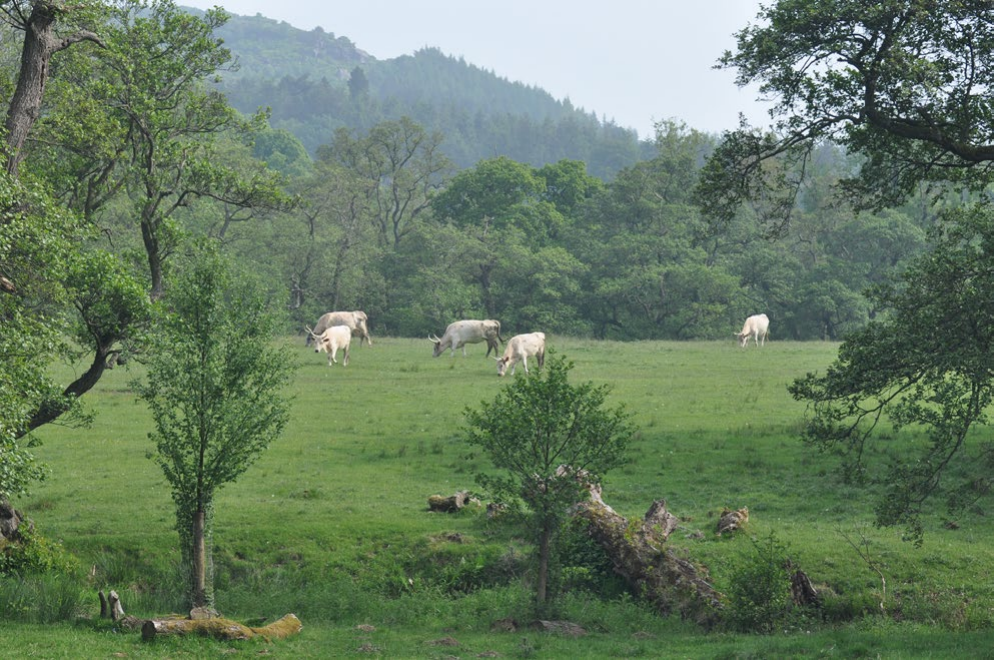
Cattle of the Chillingham herd on pasture in the lower ground of Chillingham Park, June 2018

The most remarkable feature of Chillingham Park is the herd of white, red‐eared, horned cattle (Figure 1) that have been isolated within the Park, possibly since before the first written record which is dated 1646. This breed is distinctive genetically (Orozco‐terWengel et al., 2015; Williams et al., 2015) and intense inbreeding appears to have purged harmful recessive genes (Visscher, Smith, Hall, & Williams, 2001). However, males are subfertile with very poor semen quality (T.J. Fletcher, pers. comm.). While several British landscape parks are associated today with specific herds or breeds, the historic continuity of the Chillingham herd and its relative freedom from invasive management are unique. Indeed, worldwide, relatively few cattle live in a feral state, with unmanaged herd structure and sex ratio. In Europe, the Albères, Monchina, and Mostrenca of Spain, the Cachena (Portugal), and the Betizuak (Basque country) are described as semiferal (Porter, Alderson, Hall, & Sponenberg, 2016). In the Orkney archipelago off northern Scotland, the cattle of Swona have been feral since the 1970s (Hall & Moore, 1986).

All the above‐mentioned breeds are associated with landscapes with which they share cultural significance. In situations such as HNV systems using breeds from other countries, or rewilding projects that use Heck cattle (a synthetic “re‐creation” of the aurochs; Lorimer & Driessen, 2014), or a more recent development, the “tauros” (Pettorelli et al., 2018; Richmond, Sinding, & Gilbert, 2016), cultural significance is less evident, but may develop as time progresses.

Chillingham cattle have a special official status (Hall et al., 2005), being excluded from the food chain and not required to have ear tags or passports. By special permission, routine testing of the Chillingham herd for bovine tuberculosis is replaced by autopsy of at least one animal per year. Similar provisions apply elsewhere, for example, in the Netherlands a special status of “not kept” is possible (Vermeulen, 2015).

Chillingham cattle are not housed in winter and receive hay in response to appetite. They are relatively small‐bodied (mature bull: 300–430 kg, cow approx. 280 kg); about half the body weight of the British beef breeds such as the Galloway cattle frequently used in extensive grazing systems. Practical local experience with beef cattle indicates that Chillingham Park could support a mixed‐age herd of 120 animals, provided winter feed is given (C.J. Leyland, pers. comm.) and provided there are no sheep. As a result of intensification practices aimed to secure the herbage supply of the herd and the sheep flock, plant species richness was lost between 1979 and 2008 (Bunce & Hall, 2013). The opportunity now exists to improve the general floral and faunal biodiversity of the Cattle Park by promoting recovery of plant species richness.

Management policy is to keep the herd at just over 100 total. Apart from a reserve herd in northeast Scotland, this is the only herd of the breed. In February 2019, there were about 108 cattle in the Chillingham herd with approximately equal numbers of males and females. Approximately 10 calves are born per year, during all months. Unlike in strictly seasonal animals, given adequate nutrition, there is no physiological barrier to bovine reproduction at any time of year.

There is no castration. Culling has been on the basis of euthanasia for reasons of individual welfare. From 1946 to 2009, totals of 13 animals (5 males and 8 females) of mixed ages were culled. A further 12 young animals (5 males and 12 females) were translocated to a reserve herd established in the early 1970s in Scotland. None of these animals were returned to the herd. Subsequent to 2009, the threshold for welfare culling was lowered and a further 74 animals (43 males, 31 females) of a variety of ages were culled to the end of 2017. With this new mortality factor in play, data from after 2009 are reported here, but not analyzed.

Key events in the herd since 1945 have been:

- 1947: Heavy winter mortality reduced herd from 33 total to 8 cows and 5 bulls;
- 1963: The sheep flock in the Cattle Park was increased from 180 to 300 ewes (South Country Cheviot, body weight 48 kg);
- 1980: Deaths of six lactating cows attributed to magnesium deficiency. A rotational programme of fertilization (magnesian limestone) commenced, finally discontinued in 2004 (Bunce & Hall, 2013). No lactating females have been lost for nutritional causes since that date;
- 1981: Sheep husbandry was intensified with progressive shift to crossbred ewes (73 kg body weight), flock size 300 ewes;
- 1983: Die‐off of young and senescent animals, of 27 males and 41 females present in January, 19 males and 13 females (47%) died;
- 2003–2007: progressive reduction in the sheep flock from 300 ewes to zero, followed by extinction of sheep grazing tenancy. From 2007, mechanical clearance of bracken (*Pteridium aquilinum*) was intensified;
- 2007–2017: Support for conservation work received from UK Government's Higher Level Stewardship Scheme (Rural Development Service, 2005). Continued for a further five years under the successor Higher Level Countryside Stewardship Scheme.

**Figure 2:**
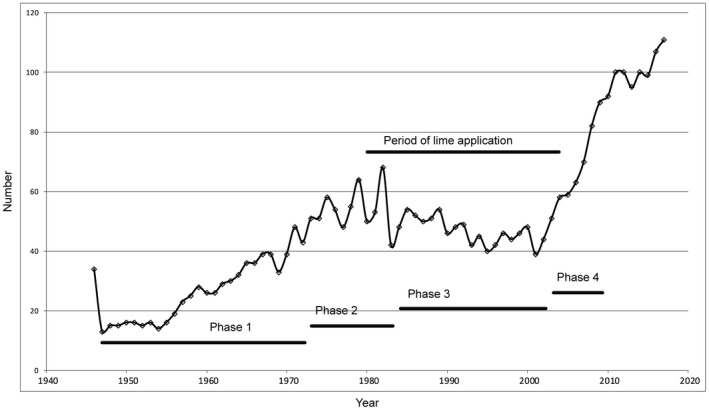
Numbers (sexes combined) of cattle in the Chillingham herd, on December 31, 1945–2017. Period of lime application (1980–2004) and the subjectively defined phases of the study are indicated (see text)

Herd numbers at 31 December each year are compared in Figure 2. Four phases can be distinguished subjectively since the 1947 collapse, on the basis of general pattern in change of numbers:

- Phase 1: steady recovery in numbers for 26 years to a total of 50 in 1973;
- Phase 2: fluctuating numbers for 11 years, reverting to 48 in 1984;
- Phase 3: slow decline of herd numbers for 18 years with signs of recovery from 2002;
- Phase 4: rapid increase in numbers from 2003;
- Current phase: herd numbers influenced by greater incidence of culling.

Biomass of the Chillingham herd (223 kg/ha; Bunce & Hall, 2013) is high compared with other relatively unmanaged cattle. In the Netherlands reserve of Kennemerduinen (2069 ha, 79 cattle) where there is no winter feeding, biomass is 17 kg/ha (Cromsigt, Kemp, Rodriguez, & Kivit, 2018). On Swona (Hall & Moore, 1986), 113 ha are occupied by 15–20 totally unmanaged cattle which numbered 17 in January 2017 (C. Annal, personal communication), a biomass of 40–88 kg/ha. The sole winter forage supply is cast‐up seaweed whose abundance varies from year to year. Generally, in Europe, in extensive husbandry with winter feeding, cattle biomass is very variable (100–500 kg/ha; Hall, 2018).

In principle, the continuance of the Chillingham herd could be achieved with a much smaller herd under a more intensive and invasive management regime but the issues of tradition, male subfertility and demographic stochasticity make this inadvisable.

## 3 MONITORING METHODS: PLANTS

Vegetation studies commenced in 1978 to support behavioral ecology studies (Hall, 1988,1989). The first plant survey used the approach which was subsequently formalized as the Countryside Vegetation System (CVS; Bunce et al., 1999). This avoids the difficulties of repeatability associated with phytosociological approaches (Hearn et al., 2011). Fifty quadrats, each of 100 m^2^, were surveyed in 1979 and in the period 2006–2008. Of these 50, 42 were resurveyed in 2017, the others being lost to tree planting. Four new quadrats were established, consequently some results were obtained from a total of 46 quadrats. Species lists with percentage estimates of cover were generated, for flowering plants, ferns, and mosses. Using CVS and MAVIS software (Carey et al., 2008), quadrats were assigned to vegetation classes. For some analyses, these were grouped into Aggregate Classes (AC). Statistical ordination places the vegetation classes on a gradient corresponding to a function of decreasing soil nutrients, increasing shade, and decreasing disturbance. The emphasis of the CVS system is on the use of plant species to achieve environmental characterizations, by assigning to the quadrats Ellenberg indicator values (four variates: acidity, fertility, light, and wetness) and overall plant strategy scores (three strategies: Competitor, Stress‐tolerator, and Ruderal). This enabled environmental changes between the years 1979, 2008, and 2017 to be visualized.

Tables of species occurrence and cover abundance were compiled. Species defined in the Countryside Stewardship 2018 Agreement Document between Natural England and CWCA, as indicators of “semi‐improved grassland” and “dry acid lowland grassland”, were distinguished.

In 1979, the vegetation of Chillingham Park was found to be, mainly, infertile, relatively species‐rich grassland of a type then still widespread in Britain (Hall & Bunce, 1984). The vegetation was resurveyed by Bunce and Hall (2013) in 2006–2008 (denoted here as 2008). Diversity was found to have declined, as would have been anticipated from the liming programme. Regular bracken cutting was undertaken from 2002 to improve the pasture resource in terms of productivity and of species richness. Some wet ground was drained in order to reduce incidence of liver fluke, and further tree regeneration and woodland enhancement were initiated.

## 4 MONITORING METHODS: CATTLE

Herd records were available for the period 1945 to December 2017. Except during limited periods animals were not individually identifiable but all known births and deaths, and sexes were recorded. Animals in at least their third year of life were defined as adults. ULM software (Legendre & Clobert, 1995) was used to calculate the asymptotic natural rate of population increase *λ* (S.J.G. Hall, unpublished).

## 5 RESULTS

### 5.1 Population dynamics

From January 1, 1945, to December 31, 2009, 251 male and 257 female calves were born. The overall mean number of calves born per year per adult female was 0.42 (*SD* 0.160, *n* = 65). The herd sex ratio (M/M + F) each December 31 averaged 0.43. Since 2000, it has ranged between 0.43 and 0.53.

Adult survival rates over the whole study are compared between the sexes and the phases of the study in Table 1. Comparisons between the phases, and female fertility rates and calculated values for *λ*, are also given.

**Table 1:** Fertility and adult survival rates of cattle in the Chillingham herd, with asymptotic natural rate of population increase *λ*, according to the phase of the study. Fertility is defined as ratio of number of calves born per adult female (at least 3 years old on January 1), averaged across the phase

Phase	Fertility	Adult female survival ≥3 years old	Adult male survival ≥3 years old	*λ*
1: 1947–1972	0.484	0.894	0.826	1.008
2: 1973–1983	0.514	0.902	0.814	1.022
3: 1984–2002	0.448	0.887	0.853	0.979
4: 2003–2009	0.448	0.932	0.946	1.039
			Mean	1.012

### 5.2 Vegetation survey

The 2017 survey assigned 36 quadrats to Aggregate Class IV (AC IV; Infertile Grassland), 1 to AC VI (Lowland Wooded), and 5 to AC VII (Upland Grasslands). The single quadrat in AC III (Fertile Grasslands) in 2008 had reverted to AC IV in 2017. The grazed area (113.09 ha) of the park in 2017 is therefore estimated as 97 ha of Infertile Grassland, 2.6 ha of Lowland Wooded, and 13.5 ha of Upland Grasslands. In 1979, the corresponding values were 70.1 ha, 13.6 ha, and 29.4 ha.

#### 5.2.1 Changes in species richness

The numbers of species of flowering plants, ferns, and mosses, per 100 m^2^ quadrat were as follows (mean, with standard deviation *SD*, and *n* in brackets) 1979:33.8 (6.52, 50); 2008:22.6 (5.09, 50); 2017:26.3 (5.38, 46). The maximum numbers of species per quadrat in the three surveys were, respectively, 44, 34, and 38 and minimum numbers 19, 10, and 18. Species occurrences and cover abundances are in Tables 2 and 3. Of the 17 species that were relatively widespread in 1979 and showed a marked decline in 2008, ten showed a further decline or no change in occurrence in 2017. The most dramatic changes were the loss of the grass *Agrostis canina*, and the mosses *Brachythecium rutabulum* and *Plagiothecium undulatum*. In terms of cover abundance, the greatest proportionate declines were of the herbs *Potentilla erecta* and *Oxalis acetosella*. The seven species that showed recovery were the grasses *Danthonia decumbens*, *Nardus stricta*, *Poa annua*, *P. pratensis*, the mosses *Rhytidiadelphus squarrosus* and *Acrocladium cuspidatum*, and the herb *Galium saxatile*.

**Table 2:** Numbers of quadrats in which selected plant species occurred that showed changes or stasis in occurrence (numbers of quadrats in which recorded) over the period 1979–2017. In 1979 and 2008, 50 quadrats were recorded; in 2017, 46. Indicators of semi‐improved grassland (see text) are in bold. Indicators of lowland dry acid grassland underlined. x: detected elsewhere in Park; blank: undetected. (a, b): Species that were widespread in 1979 with substantial change in occurrence to 2008; (c): other species with marked change from 2008 to 2017; (d): species that stayed relatively unchanged; (e): other indicator species for semi‐improved grassland and for lowland dry acid grassland

	1979	2008	2017
(a) Species with >10 records in 1979 and a decline >50% in 2008, further decline or no change in 2017			
*Agrostis canina*	14		
*Brachythecium rutabulum*	12	4	
***Cardamine pratensis***	21	5	5
*Conopodium majus*	27	4	1
*Festuca ovina*	43	x	1
*Luzula multiflora*	36	11	4
*Oxalis acetosella*	22	9	7
*Phleum pratense*	19	2	1
*Plagiothecium undulatum*	17		
*Veronica officinalis*	13		1
(b) Species with >10 records in 1979 and a decline of more than 50% in 2008, with increase in 2017			
*Acrocladium cuspidatum*	11		6
*Danthonia decumbens*	19	1	4
*Galium saxatile*	35	1	5
*Nardus stricta*	14	3	7
*Poa annua*	31	1	3
*Poa pratensis*	33	8	9
*Rhytidiadelphus squarrosus*	35	10	18
(c) Other species that have shown a marked change from 2008 to 2017			
*Cirsium vulgare*	9	17	5
*Deschampsia cespitosa*	47	42	34
*Euphrasia officinalis*	3	4	14
***Hypochaeris radicata***		1	10
*Juncus effusus*	31	29	18
*Lathyrus pratensis*	7	8	2
***Leontodon autumnalis***	1	4	10
*Poa trivialis*	36	30	7
*Potentilla sterilis*	7	5	14
*Senecio jacobaea*	x	2	11
*Stellaria graminea*	23	25	14
*Taraxacum agg*.	15	15	25
***Trifolium pratense***	3	1	13
*Trisetum flavescens*	4	13	2
(d) Species with >10 records in 1979, 2008 and 2017			
*Agrostis tenuis/capillaris*	47	45	45
*Anthoxanthum odoratum*	49	45	46
*Cerastium fontanum*	35	35	37
*Cirsium arvense*	29	29	35
*Cirsium palustre*	18	25	23
*Cynosurus cristatus*	31	38	38
*Dactylis glomerata*	14	22	21
*Festuca rubra*	41	47	45
*Holcus lanatus*	50	46	44
*Holcus mollis*	11	15	16
*Juncus articulatus*	23	20	17
*Lolium perenne*	16	26	28
*Lotus corniculatus*	19	15	13
***Plantago lanceolata***	23	28	32
*Potentilla erecta*	46	40	38
***Prunella vulgaris***	15	20	24
*Pteridium aquilinum*	31	34	32
***Ranunculus acris***	28	26	31
*Ranunculus repens*	25	28	27
***Rumex acetosa***	42	41	39
*Trifolium repens*	35	42	44
***Veronica chamaedrys***	32	24	23
*Viola riviniana*	36	21	18
(e) Records of indicator species not listed above			
***Achilllea millefolium***	5	x	3
*Anemone nemorosa*		x	
*Campanula rotundifolia*	1		1
*Galium saxatile*	35	1	4
*Lathyrus montana*	x		x
*Pedicularis sylvatica*	x		
*Polygala spp.*	4		
***Potentilla reptans***	x		
*Rumex acetosella*		x	
*Vaccinium myrtillus*	6	x	
***Vicia cracca***			1

**Table 3:** Cover abundance of flowering plants and bracken *Pteridium aquilinum* over entire park. Rankings are highly correlated between years (Kendall W test: chi2 = 94.67, *p* < 0.001). Indicators of semi‐improved grassland (see text) are in bold. Indicators of lowland dry acid grassland are underlined

Species in descending order of cover in 1979	1979	2008	2017
*Pteridium aquilinum*	40.12	31.54	11.04
*Holcus lanatus*	28.40	29.24	15.72
*Agrostis capillaris*	22.78	43.62	37.00
*Potentilla erecta*	17.78	6.26	1.20
*Deschampsia cespitosa*	13.14	9.26	9.46
*Festuca rubra agg*.	12.88	38.98	11.98
*Trifolium repens*	11.38	16.89	8.15
*Anthoxanthum odoratum*	11.32	11.94	12.43
*Juncus articulatus*	7.16	5.54	1.72
*Oxalis acetosella*	6.88	1.74	0.15
*Cynosurus cristatus*	6.82	16.24	6.80
*Juncus effusus*	5.64	2.96	4.28
*Cirsium arvense*	5.56	4.56	7.35
*Poa pratensis sens.lat*.	5.34	0.24	0.83
*Viola riviniana*	5.24	1.72	0.39
*Lolium perenne*	4.06	9.46	8.89
*Nardus stricta*	3.06	0.16	0.80
***Rumex acetosa***	2.76	3.08	1.02
*Poa annua*	2.54	0.02	0.07
*Holcus mollis*	1.88	6.18	0.61
*Dactylis glomerata*	1.82	6.34	2.02
*Poa trivialis*	1.80	1.14	0.15
***Veronica chamaedrys***	1.78	1.10	0.50
*Danthonia decumbens*	1.78	0.02	0.09
***Plantago lanceolata***	1.68	3.14	2.65
*Phleum pratense sens.lat*.	1.66	0.90	0.02
*Cerastium fontanum*	1.48	1.42	0.80
*Ranunculus repens*	1.28	1.78	0.78
***Ranunculus acris***	1.24	1.26	0.85
*Urtica dioica*	1.16	1.12	1.04
*Conopodium majus*	1.12	0.08	0.02
*Lotus corniculatus*	1.04	0.74	0.48
***Cardamine pratensis***	0.86	0.10	0.11
***Prunella vulgaris***	0.72	1.40	0.70
*Cirsium palustre*	0.58	0.50	0.59
*Taraxacum agg*.	0.42	0.48	0.54
*Cirsium vulgare*	0.40	0.48	0.11
*Trisetum flavescens*	0.20	0.26	0.04
*Carex flacca*	0.20	3.40	1.22

When the 2017 quadrats were grouped into Aggregate Classes, mean species richness was, for AC IV, AC VI, and AC VII, respectively, 26, 21, and 31.

#### 5.2.2 Changes in indicator species

Of 23 indicators of semi‐improved grassland and 38 of lowland dry acid grassland, 12 and 14, respectively, have been recorded in our study (of which ten and eight, respectively, in 2017). Of the 18 indicators noted in 2017, 13 showed an increase or no change in number of quadrats where they were found (Table 2). Of the ten indicators for which total cover abundance was assessed all except the herb, *Cardamine pratensis* showed a decline from 2008 to 2017 (Table 3).

Bracken (*P. aquilinum*) showed a substantial decrease in cover abundance from 2008 to 2017 because of mowing (Kruskal–Wallis test for difference of medians *χ* ^2^ = 8.445, *df* = 2, *p* = 0.0147; Table 4). Percentage reduction in bracken cover was not significantly correlated with absolute increase in number of species (*n* = 29 quadrats, *r* = 0.28; the only species to show a marked increase in cover abundance was the Competitor (Grime, Hodgson, & Hunt, 2007) species *Cirsium arvense*.

**Table 4:** Distribution of bracken (*Pteridium aquilinum*). % cover values were not normally distributed

	1978	2008	2017
Number of quadrats surveyed	50	50	46
Number with bracken cover <5%	20	17	24
Number with bracken cover >5%	30	32	22
Median cover	39	20	5
Maximum cover	100	100	60
Minimum cover	0	0	0

#### 5.2.3 Environmental changes

**Figure 3:**
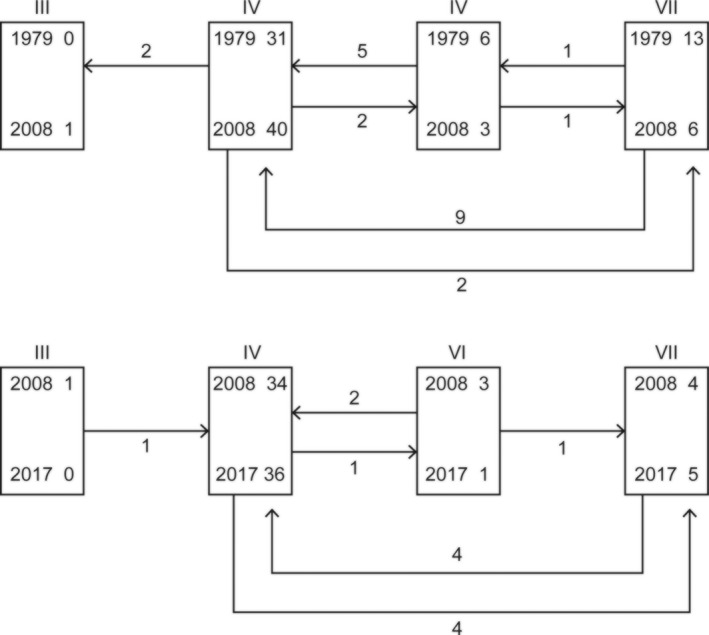
Shifts between Aggregate Classes (AC) from 1978 to 2008, and from 2008 to 2017. Numbers within boxes signify numbers of quadrats assigned to each AC at each survey. Numbers associated with arrows indicate numbers of quadrats that shifted from one AC to another, in the period between the two years indicated. In 1979 and 2008, 50 quadrats were surveyed in each year. Of these 50, 42 were resurveyed in 2017

**Figure 4:**
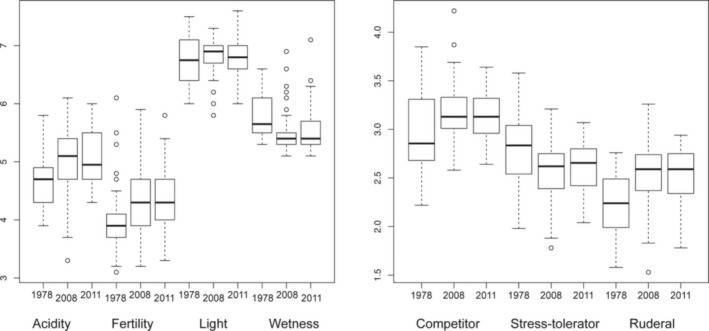
Changes from 1979 to 2008 and 2008 to 2017 in Ellenberg scores and on the competitor/stress‐tolerator/ruderal scales (see text). Kruskal–Wallis tests for heterogeneity of medians, for each trio of boxplots, were all significant (*p* < 0.01) except for that relating to “light”. All post hoc comparisons between 1978 and 2008 boxplots gave significant results, while none of the 2008–2017 comparisons did so (details in text)

The MAVIS analysis showed that, at the level of Aggregate Classes, there was very little overall change between 2008 and 2017 (Figure 3). From 1979 to 2008, there had been considerable change of AC VI Lowland Wooded and AC VII Upland Grasslands into AC IV Infertile Grasslands. This is also evident from changes in Ellenberg and C/S/R scores (Figure 4). For all seven groups of boxplots in Figure 4, the Kruskal–Wallis test indicated significant differences between years. Post hoc paired, one‐tailed Wilcoxon tests showed that all of the apparent differences between 1978 and 2008 were significant (*p* < 0.001). None of those between 2008 and 2017 were significant. Acidity appears to have shown a small increase from 2008 to 2017, but the difference is not significant (*p* = 0.193).

## 6 DISCUSSION

In many respects the Chillingham cattle and their parkland habitat are unique, and while this is a reason to develop an evidence base to underpin their conservation, the results of such research need to be transferable. Long‐term monitoring of the herd has already shown this by providing some important scientific insights. Comparison of birth and death data from the mid‐19th century with those from the late 20th century (Hall & Hall, 1988) demonstrated continued viability, and the reproductive rate is now higher than when it was last studied intensively (1953–1985). This has implications for the genetic understanding of inbreeding (Visscher et al., 2001; Williams et al., 2015). Also, analysis of birth dates collected since 1946 yielded one of the very few mammalian examples of phenological effects of climate warming (Burthe, Butler, Searle, Hall, & Thackeray, 2011). If the distribution of birth dates over the year is expressed in relation to a fixed date, the median date of conception is seen to have advanced by one day per year over the period 1947–2008, from late September to late July. This phenological change is ascribed to climate warming, acting through advance of the herbage growing season.

We report two new sets of results, firstly, the recent changes in population dynamics of the herd (probably influenced by the greater abundance of herbage following the removal of sheep), and secondly, documentation of vegetation change whose causes, due to the lack of controlled experimentation, we cannot ascertain (including liming, sheep grazing, and eutrophication by nitrogen deposition).

### 6.1 Population biology of free‐living cattle

Our findings on population dynamics provide new characterization information on free‐living cattle. This will help to remedy what Pettorelli et al. (2018) describe as “a perceived lack of empirical information [relevant to rewilding] … much could be achieved by adequately synthesizing existing information.” Ours is the only long‐term dataset on the population dynamics and behavioral ecology of relatively unmanaged cattle of natural sex ratio and age distribution.

Since 2003, the rate of population growth (*λ*) of 1.039 has been rather lower than those observed with wild‐living Holarctic bovines (Yellowstone bison *λ* = 1.07, Fuller et al., 2007; wisent *λ* = 1.049–1.152, Mysterud et al., 2007). In Oostvaardersplassen, the Heck cattle introduced in 1983 numbered over 500 in 2000 (*λ* = 1.189, calculated from Cornelissen, Bokdam, Sykora, and Berendse (2014). With a herd totaling over 100 cattle of which half are males, and producing around 10 calves per year, the Chillingham system has little in common with commercial farming. Yet, the high number of males is necessary because of their poor fertility. The spacing behavior of adult bulls (Hall, 1988) has implications for the spread of grazing behavior around the park, though whether this influences vegetation patterns over time has not been studied.

In most years, mortality at Chillingham is relatively light; there have been occasional die‐offs though these have been much less dramatic than those observed with, for example, the Soay sheep of St. Kilda or the red deer (*Cervus elaphus*) of Rum. These populations often exhibit sizeable die‐offs; over 50% in one in five years for the sheep, and one in six years for the deer, and for both populations die‐offs of over 30% have been recorded in over 40% of years (ICMO, 2006). At Chillingham, die‐offs of over 30% have been observed in only two years since 1947 and management policy is to avoid these as they could raise concerns for animal welfare, as has happened at Oostvaardersplassen (Gamborg, Gremmen, Christiansen, & Sandøe, 2010). There is no evidence of density dependence in mortality or birth rate (present study, and Hall & Hall, 1988).

Patterns of change in cattle numbers suggest that the liming programme and the resulting vegetation changes, which were evident in 2008 (Bunce & Hall, 2013) were of limited benefit to the cattle which showed a decline in numbers over the period. The sheep may have prevailed in competition for the available herbage, as predicted by Illius and Gordon (1987). The implication is of a conflict of interest between the cattle and the commercial sheep flock, which was only resolved by the purchase by CWCA of the grazing lease and the removal of the flock.

### 6.2 Chillingham cattle and their botanical and cultural environments

The priority at Chillingham continues to be the maintenance of a cattle breed of national significance, and there is now evidence of some rectification of the damage caused to floral biodiversity by past management practices. The importance is also highlighted, of practical and scientifically sound long‐term monitoring, while the vegetation studies outlined here represent one of the very few long‐term (1978–2017) narratives on the interaction between holarctic megaherbivores and their forage resource. The cultural landscape of Chillingham Park can be summarized as a designed early 19th‐century park imposed on a medieval wood pasture (Hall, 2013). Its relationship to local history and other cultural features of the area are discussed in Bahn and Mutimer (2016).

Within the classification system of the Countryside Survey, the principal vegetation type of the Chillingham Park pasture is Aggregate Class IV Infertile Grasslands (AC IV). Species richness of this AC in Britain showed a reduction from 20.1 to 19.3 species per 200 m^2^ quadrat over the period 1998–2007 Carey et al. (2008). At Chillingham, AC IV is therefore comparatively species‐rich, with more species in a smaller quadrat (25.7 species per 100 m^2^ quadrat) and is therefore an important resource of this biodiverse habitat.

Mitchell et al. (2017) found that in Scottish grasslands over the period 1973–2013, “dominant plant species” increased in cover, apparently showing an increase in the rankness of vegetation, reflecting a decrease in grazing since the 1990s. In Chillingham Park, the reduction in bracken from 1979 to 2008 appeared to result in the spread of *Agrostis capillaris* and *Festuca rubra*, with emergence of *Cynosurus cristatus* and *Trifolium repens*, two notably light‐requiring species (Grime et al., 2007). Since 2008, the considerable reduction in cover abundance of bracken has not yet resulted in particular species taking over an ecologically dominant role. An increased accumulation of litter is implied, though data are lacking.

At the level of Aggregate Classes, there was no net change from 2008 to 2017. Ellenberg values do not show statistically significant changes from 2008 to 2017, toward the values that had been obtained in 1979, but the overall pattern implies that the loss of species diversity has been arrested. Changes in the distribution of indicator species could also imply that recovery of species richness is under way.

The liming programme, deposition of anthropogenic fixed nitrogen, bracken control, and grazing by sheep all probably interacted to cause the vegetation changes observed from 1979 to 2008. Effects are likely to be long‐lasting (Melts et al., 2018). For example, in arable land in Canada, the effect of a single application of lime on soil pH was still detectable 30 years later (Beckie & Ukrainetz, 1996). Some changes in species cover and abundance suggest a decrease in fertility from 2008 to 2017; changes in quadrat from one vegetation class to another are consistent with this, but statistically significant effects are not present.

### 6.3 Multifunctionality in conservation

The Chillingham study is the first published example of multiple conservation benefits coming from a plant–herbivore system operating in an acknowledged cultural and historic landscape. Chillingham Park exemplifies a medium productivity environment that would be best managed with a land‐sharing, rather than land‐sparing approach (Maskell et al., 2013), and is an expression of the linkage that is envisaged between agri‐environment schemes and ecosystem services (Whittingham, 2011). In practical terms it highlights the relative ease of long‐term monitoring and, together with earlier publications (for example, Hall, 1989; Hall & Hall, 1988) provides baseline data on the biology of nonhusbanded cattle, of potential value for rewilding projects. Management priority will continue to be assigned to the cattle, but the value of Chillingham Park as a species‐rich example of a diminishing habitat type is now acknowledged.

That the conservation benefits are multiple at Chillingham arises from the cattle being of a recognized pure, native breed. While this will not be feasible in all habitat management situations, we recommend that the use of traditional breeds, if possible those of local cultural significance, should be carefully considered. Reasons commonly given for a cattle breed being particularly suited for habitat management include low selectivity, wide spacing during grazing, tolerance of biting insects, and general hardiness. These characteristics are not easily researchable; formal experimental studies have focussed on aspects of foraging behavior, and have not provided strong evidence of differences purely attributable to breed, emphasizing the probable importance of body size and the prior experience of the animals (Orr, Tallowin, Griffith, & Rutter, 2014; Rook et al., 2004). Local breeds may, however, be unavailable or considered unsuitable for this purpose and in practice, small‐bodied beef breeds such as the Galloway and Highland or the more traditional form of the Hereford (all of which have daughter breed societies in many other countries: Porter et al., 2016) have been favoured. We emphasize that appropriate breed choice will add to the conservation benefits of a habitat management scheme.

Breed choice for trophic rewilding will be more restricted, with a premium being on hardiness, but the persistence of the Swona herd (descendants of Shorthorn ‐ Aberdeen Angus crossbreds) in apparently difficult conditions suggests the choice may be fairly wide. Heck cattle or the “tauros” would be other choices, which could be controversial for reasons beyond the scope of this paper.

In Europe, cattle *B. taurus* are subject to national animal welfare and health laws which can, however, be adapted to accommodate free‐living herds (Hall et al., 2005; Vermeulen, 2015). There is much public interest in rewilding (Jorgensen, 2015) and considerable public sensitivities to the welfare of extensively kept cattle (van Klink & Kampf, 2008), which will need to be addressed early in project development.

### 6.4 Livestock biodiversity in a broader context

A prediction of the present discussion that could be tested by modeling is that the overall monetized and nonmonetized conservation benefits of the management of HNV systems can be enhanced by the use of traditional livestock breeds.

Livestock biodiversity has clear intrinsic value as cultural and social assets well as economic value as a genetic resource, while its cultural value can also yield economic benefit in the form of local food and other specialities. It is enfolded into human culture, which Jorgensen (2015) emphasizes would be at risk in conservation models which seek to remove the human element from environments. There are many areas in Europe where society is not ready to accept what is seen as full rewilding of postproductive and abandoned landscapes, but where “nature‐and‐culture‐friendly” land uses, based on the husbandry of traditional livestock breeds and the securing of floral and faunal biodiversity, could be acceptable.

## CONFLICT OF INTEREST

None declared.

## AUTHOR CONTRIBUTIONS

Both authors conceived the ideas. SJGH collated and analyzed the population data recorded by the Chillingham Wild Cattle Association, of which he is Vice Chairman. RGHB coordinated the vegetation studies. SJGH and RGHB wrote the manuscript. Both authors contributed critically to the drafts and gave final approval for publication.

## Data Availability

Data are in the Dryad Digital Repository https://doi.org/10.5061/dryad.2gm8348.
